# A retrospective study of metaplastic breast cancer: Treatment and prognosis through chemotherapy followed by radiotherapy

**DOI:** 10.1097/MD.0000000000049401

**Published:** 2026-08-07

**Authors:** Zankai Wu, Yanting Zhang, Yun Bao, Lingxia Liao, Yiping Gong, Jin Hu

**Affiliations:** a Department of Breast and Thyroid Surgery, Renmin Hospital of Wuhan University, Wuhan, Hubei, China; b Department of Ultrasound Medicine, Union Hospital, Tongji Medical College, Huazhong University of Science and Technology, Wuhan, Hubei, China; c Department of Anesthesiology, Renmin Hospital of Wuhan University, Wuhan, Hubei, China.

**Keywords:** breast cancer, chemotherapy, metaplastic, prognosis, radiotherapy

## Abstract

Metaplastic breast cancer (MBC) is a rare and aggressive form of breast cancer. The effectiveness of chemotherapy followed by radiotherapy (CFRT) for MBC remains controversial. The present study aimed to evaluate the efficacy of CFRT on MBC patients with N0M0. A retrospective study was performed to analyze MBC from the surveillance, epidemiology, and end results database. Breast cancer-specific survival (BCSS) and overall survival (OS) rates were analyzed using the Kaplan–Meier curve. Cox proportional hazard models were used to assess BCSS and OS. CFRT might be a valid therapeutic strategy for MBC with N0M0, especially considered with surgical planning. Of the 4672 MBC patients in the surveillance, epidemiology, and end results registry, our final sample comprised 2260 patients. A total of 602 (26.6%), 292 (12.9%), 638 (28.2%), and 728 (32.2%) patients received non-therapy, only-radiotherapy, only-chemotherapy, and CFRT, respectively. Age, tumor size, tumor grade, surgery, and therapy types were independent indicators for BCSS. The Kaplan–Meier analysis showed that total mastectomy (TM) not breast-conserving surgery (BCS) improved BCSS of MBC receiving CFRT, whereas OS of MBC receving CFRT can not benefit from surgical type. Further exploration illustrated that, for BCSS, only stage T2 and T3 MBC patients underwent BCS and only stage T3 MBC patients underwent TM could benefit from CFRT. However, for OS, all stage MBC patients underwent BCS and only stage T3 MBC patients underwent TM could benefit from CFRT.

## 1. Introduction

Metaplastic breast cancer (MBC) is an extremely rare and aggressive^[1,2]^ form of the disease, making up < 1% of all breast cancers.^[3,4]^ Metaplastic histology was identified as a unique pathological type by the World Health Organization in 2000.^[5]^ This type of cancer is characterized by various subtypes such as carcinosarcoma, spindle cell carcinoma, squamous cell carcinoma, matrix-producing carcinoma, and carcinoma with osteoclastic giant cells.^[6–10]^

Of note, the management of MBC is still paralleled to that of invasive ductal carcinoma (IDC), according to the breast cancer guidelines by the National Comprehensive Cancer Network.^[11]^ However, MBC, tends to have higher tumor grades, larger tumor sizes, and less regional node metastasis compared to breast cancers with more common histology.^[12–14]^ Studies have shown that MBC patients with stage I to III disease had significantly worse 5-year breast cancer-specific survival (BCSS) compared with synchronous IDC.^[15]^ Previous studies illustrated that MBC is chemically refractory to neoadjuvant and adjuvant therapy.^[2,16–18]^

Although previous studies had suggested that MBC patients might not benefit from chemotherapy (CT).^[2,13,15,16]^ At the genetic level, MBCs are characterized by recurrent mutations affecting TP53 and genes related to the PI3K/AKT/mTOR, MAPK, Wnt, and Notch signaling pathways,^[19–22]^ explaining its chemo-resistance. Moreover, the effectiveness of radiotherapy (RT) for MBC remains controversial.^[13,23]^ Multimodality treatment, or CT followed by RT (CFRT), is the standard treatment for breast carcinoma. Most studies have focused on the effects of adjuvant RT or CT alone on long-term outcomes for patients with MBC. However, few well-performed studies have been conducted on the efficacy of CFRT in patients with resectable MBC.

## 2. Materials and methods

### 2.1. Surveillance, epidemiology, and end results (SEER) database and patients

According to the diagnostic code of the International Classification of Diseases for Oncology, Version 3, malignant tumors were recorded in the SEER database which consists of open access data from 18 population-based cancer registries in the United States. In our study, the International Classification of Diseases for Oncology, Version 3 codes included 8560, 8562, 8570 to 8572, 8575, and 8980 to 8982.^[24–27]^ We first identified 4163 patients diagnosed with MBC between 2000 and 2016. After sequential exclusions; men (n = 6), non-first primaries (n = 351), bilateral tumors (n = 2), cases lacking histologic confirmation (n = 1) or survival data/autopsy-only diagnosis (n = 81), T0/Tis lesions (n = 6), non-N0 stage (n = 1024), non-M0 stage (n = 221), T4 or unknown T-stage (n = 104) and missing CT or RT records (n = 107), 2260 patients remained for the final analysis. (Table 1)

**Table 1:** Stepwise inclusion and exclusion counts.

Removal criterion	Removed	Remaining
2000–2016 MBC patients	360	4163
Exclude men	6	4157
Exclude patients younger than 18 yrs	0	4157
Exclude patients without histology or cytology confirmation	1	4156
Exclude patients whose tumor was not the first tumor	351	3805
Exclude patients with bilateral involvement	2	3803
Exclude patients without survival information/diagnosed by autopsy/ death record only	81	3722
Exclude patients whose disease is stage T0/Tis	6	3716
Exclude patients whose disease is not stage N0	1024	2692
Exclude patients whose disease is not stage M0	221	2471
Exclude patients whose disease is stage T1–3	104	2367
Exclude patients without information of chemotheropy and radiotherapy	107	2260
Final data set		2260

MBC = metaplastic breast cancer.

### 2.2. Demographic and clinicopathologic features

Our study enrolled demographic parameters, including age at diagnosis, race (White, Black, Others), and insurance, and clinicopathologic parameters, including tumor grade, histologic subtype, tumor size, regional lymph node status (N0), RT, and local therapies. The primary clinical outcome was BCSS from the date of diagnosis to the date of death caused by MBC for this series. The secondary clinical outcome in our study was the overall survival (OS) which defined from the date of diagnosis to the date of death for any cause.

### 2.3. Statistical analysis

The clinicopathological characteristics between groups were compared by the Student *t*-test and *χ*^2^ test. Using the log-rank test to compared the Kaplan–Meier model among groups for the BCSS. Univariate and multivariate Cox proportional hazards regression model was performed to evaluate the risk factors for BCSS and OS. Hazard ratios (HRs) were shown with a 95% confidence interval (CI). All statistical analyses used SPSS statistical software (version 24.0; IBM Corporation).

### 2.4. Ethics statement

This study was exempt from the approval processes of the Institutional Review Boards because the SEER database patient information is de-identified. Also, a patient consent form was not applicable.

## 3. Results

### 3.1. Demographic and clinical characteristics

A total of 4672 patients diagnosed with MBC were extracted; our final sample included 2260 cases. The demographic and clinical characteristics of the study are shown in Table 2. A total of 602 (26.6%), 292 (12.9%), 638 (28.2%), and 728 (32.2%) patients received non-therapy, only-RT, only-CT, and CFRT, respectively. The median age at diagnosis was 72 (range, 22–89 years), 72 (range, 27–89 years), 58 (range, 24–88 years), and 57 (range, 25–87 years) in the non-therapy, only-RT, only-CT, and CFRT groups, respectively. CFRT trended to be performed in patients who were younger, had poorly differentiated tumors, had larger tumor sizes, and received breast-conserving surgery (BCS).

**Table 2:** Characteristics in MBC patients with N0M0.

Variables	Non-therapy	Only-RT	Only-CT	CFRT	*P*
Age (median, range)	72 (22–89)	72 (27–89)	58 (24–88)	57 (25–87)	< .001
Age group					< .001
≤ 60 yrs	148 (24.6)	64 (21.9)	360 (56.4)	451 (62.0)	
> 60 yrs	454 (75.4)	228 (78.1)	278 (43.6)	277 (38.0)	
Survival months (median)	40.5 (1–197)	55 (1–195)	43.5 (1–194)	57 (1–202)	< .001
Race (n, %)					.002
White	492 (81.7)	234 (80.1)	487 (76.3)	541 (74.3)	
Black	68 (11.3)	42 (14.4)	91 (14.3)	137 (18.8)	
Others	42 (7.0)	16 (5.5)	60 (9.4)	50 (6.9)	
Insurance (n, %)					.001
No	192 (31.9)	98 (33.6)	148 (23.2)	208 (28.6)	
Yes	410 (68.1)	194 (66.4)	490 (76.8)	520 (71.4)	
Grade (n, %)					< .001
Well differentiated	45 (7.5)	31 (10.6)	16 (2.5)	17 (2.3)	
Moderately differentiated	92 (15.3)	65 (22.3)	67 (10.5)	61 (8.4)	
Poorly differentiated	353 (58.6)	137 (46.9)	445 (69.7)	533 (73.2)	
Undifferentiated	23 (3.8)	7 (2.4)	29 (4.5)	35 (4.8)	
Unknown	89 (14.8)	52 (17.8)	81 (12.7)	82 (11.3)	
Histology (n, %)					< .001
Metaplastic carcinoma	475 (78.9)	230 (78.8)	553 (86.7)	633 (87.0)	
Adenosquamous carcinoma	41 (6.8)	27 (9.2)	23 (3.6)	16 (2.2)	
Carcinosarcoma	20 (3.3)	4 (1.4)	29 (4.5)	29 (4.0)	
Others	66 (11.0)	31 (10.6)	33 (5.2)	50 (6.9)	
Tumor size (n, %)					< .001
T1	190 (31.6)	152 (52.1)	154 (24.1)	197 (27.1)	
T2	301 (50.0)	113 (38.7)	375 (58.8)	359 (49.3)	
T3	111 (18.4)	27 (9.2)	109 (17.1)	172 (23.6)	
Surgery (n, %)					< .001
Non-surgery	23 (3.8)	3 (1.0)	25 (3.9)	4 (0.5)	
BCS	179 (29.7)	242 (82.9)	165 (25.9)	511 (70.2)	
TM	400 (66.4)	47 (16.1)	448 (70.2)	213 (29.3)	

BCS = breast-conserving surgery, CFRT = chemotherapy followed by radiotherapy, CT = chemotherapy, MBC = metaplastic breast cancer, n = number of patients, RT = radiotherapy, TM = total mastectomy.

### 3.2. Prognostic factors for BCSS in MBC patients with N0M0

Univariate analysis showed that those parameters were associated with BCSS, including age, tumor grade, tumor size, surgery, and therapy types. A published study showed White people with MBC had a better prognosis than Black ones.^[25,27]^ Youssef et al^[28]^ reported that having insurance was associated with improved survival. Moreover, Tseng et al^[29]^ reported that factors increasing the risk of disease-related death included carcinosarcoma histology. To eliminate confounding factors, variables with clinical value and with a *P* value < .1 were included in the multivariate analysis model.

Using the multivariate Cox proportional hazards model further analyzed the independent prognostic factors associated with BCSS. Older patients had a worse prognosis (HRs: 1.014; 95% CI:1.005–1.023; *P* = .002). Therapy types (non-therapy as reference, only-RT, HRs 0.806, 95% CI 0.547–1.186, *P* = .273; only-CT, HRs 0.679, 95% CI 0.499–0.925, *P* = .014; CFRT, HRs 0.646, 95% CI 0.476–0.878, *P* = .005) and tumor size (T1 as reference, T2 HRs 1.918, 95% CI 1.388–2.649, *P* < .001; T3 HRs 5.588, 95% CI 3.928–7.949, *P* < .001) were independent prognostic factors related to BCSS. In addition, for MBC patients with N0M0, surgery was also significantly associated with better prognosis (non-surgery as reference, BCS HRs 0.329, 95% CI 0.185–0.584, *P* < .001; total mastectomy (TM) HRs 0.379, 95% CI 0.221–0.649, *P* < .001). Furthermore, tumor grades (undifferentiated as reference, poorly differentiated HRs 0.566, 95% CI 0.384–0.835, *P* = .004; moderately differentiated HRs 0.376, 95% CI 0.218–0.647, *P* < .001; well differentiated HRs 0.454, 95% CI 0.209–0.986, *P* = .046) were independent indicators of worse BCSS. (Table 3)

**Table 3:** Prognostic factors for BCSS in MBC patients with N0M0.

Variables	Univariate analysis		*P*	Multivariate analysis		*P*
	HRs	95% CI		HRs	95% CI	
Age	1.017	1.009–1.025	< .001	1.014	1.005–1.023	.002
Race/ethnicity (n, %)						
Black	1	[Reference]		1	[Reference]	
White	1.012	0.744–1.376	.938	1.064	0.778–1.456	.697
Other	0.879	0.531–1.457	.618	0.991	0.594–1.651	.972
Insurance (n, %)						
No	1	[Reference]		1	[Reference]	
Yes	1.079	0.859–1.355	.514	0.993	0.784–1.259	.955
Grade (n, %)						
Undifferentiated	1	[Reference]		1	[Reference]	
Poorly differentiated	0.502	0.342–0.736	< .001	0.566	0.384–0.835	.004
Moderately differentiated	0.304	0.108–0.514	< .001	0.376	0.218–0.647	< .001
Well differentiated	0.291	0.142–0.596	.001	0.454	0.209–0.986	.046
Unknown	0.652	0.420–1.010	.055	0.569	0.362–0.893	.014
Histology (n, %)						
Metaplastic carcinoma	1	[Reference]		1	[Reference]	
Adenosquamous carcinoma	0.545	0.2909–1.024	.059	0.767	0.387–1.520	.448
Carcinosarcoma	1.515	0.941–2.439	.087	1.227	0.757–1.990	.407
Others	0.692	0.456–1.050	.084	0.697	0.454–1.070	.990
Tumor size (n, %)						
T1	1	[Reference]		1	[Reference]	
T2	1.943	1.420–2.660	< .001	1.918	1.388–2.649	< .001
T3	5.590	3.981–7.850	< .001	5.588	3.928–7.949	< .001
Surgery (n, %)						
Non-surgery	1	[Reference]		1	[Reference]	
BCS	0.229	0.134–0.392	< .001	0.329	0.185–0.584	< .001
TM	0.424	0.251–0.716	< .001	0.379	0.221–0.649	< .001
Therapy types						
Non-therapy	1	[Reference]		1	[Reference]	
Only-RT	0.599	0.415–0.866	.006	0.806	0.547–1.186	.273
Only-CT	0.661	0.497–0.879	.004	0.679	0.499–0.925	.014
CFRT	0.677	0.519–0.883	.004	0.646	0.476–0.878	.005

BCS = breast-conserving surgery, BCSS = breast cancer-specific survival, CFRT = chemotherapy followed by radiotherapy, CI = confidence interval, CT = chemotherapy, HR = hazard ratio, MBC = metaplastic breast cancer, n = number of patients, RT = radiotherapy, TM = total mastectomy.

Simultaneously, we analyzed the independent prognostic factors associated with OS by using the multivariate Cox proportional hazards model. Some factors were determined to be independent indicators of worse OS, including age of diagnosis (HR: 1.036; 95% CI:1.028–1.034; *P* < .001), tumor grade (undifferentiated as reference, poorly differentiated HRs 0.609, 95% CI 0.444–0.836, *P* = .002; moderately differentiated HRs 0.564, 95% CI 0.384–0.829, *P* = .004; well differentiated HRs 0.427, 95% CI 0.237–0.772, *P* = .005), tumor size (T1 as reference, T2 HRs 1.683, 95% CI 1.270–2.230, *P* < .001; T3 HRs 2.412, 95% CI 1.474–3.946, *P* < .001), surgery (non-surgery as reference, BCS HRs 1.052, 95% CI 0.866–1.276, *P* = .611; TM HRs 3.454, 95% CI 2.167–5.506, *P* < .001), and therapy types (non-therapy as reference, only-RT, HRs 0.701, 95% CI 0.539–0.911, *P* = .008; only-CT, HRs 0.604, 95% CI 0.479–0.763, *P* < .001; CFRT HRs 0.501, 95% CI 0.395–0.635, *P* < .001). (Table S1, Supplemental Digital Content 1)

### 3.3. Survival analyses in patients with N0M0

**Figure 1.**
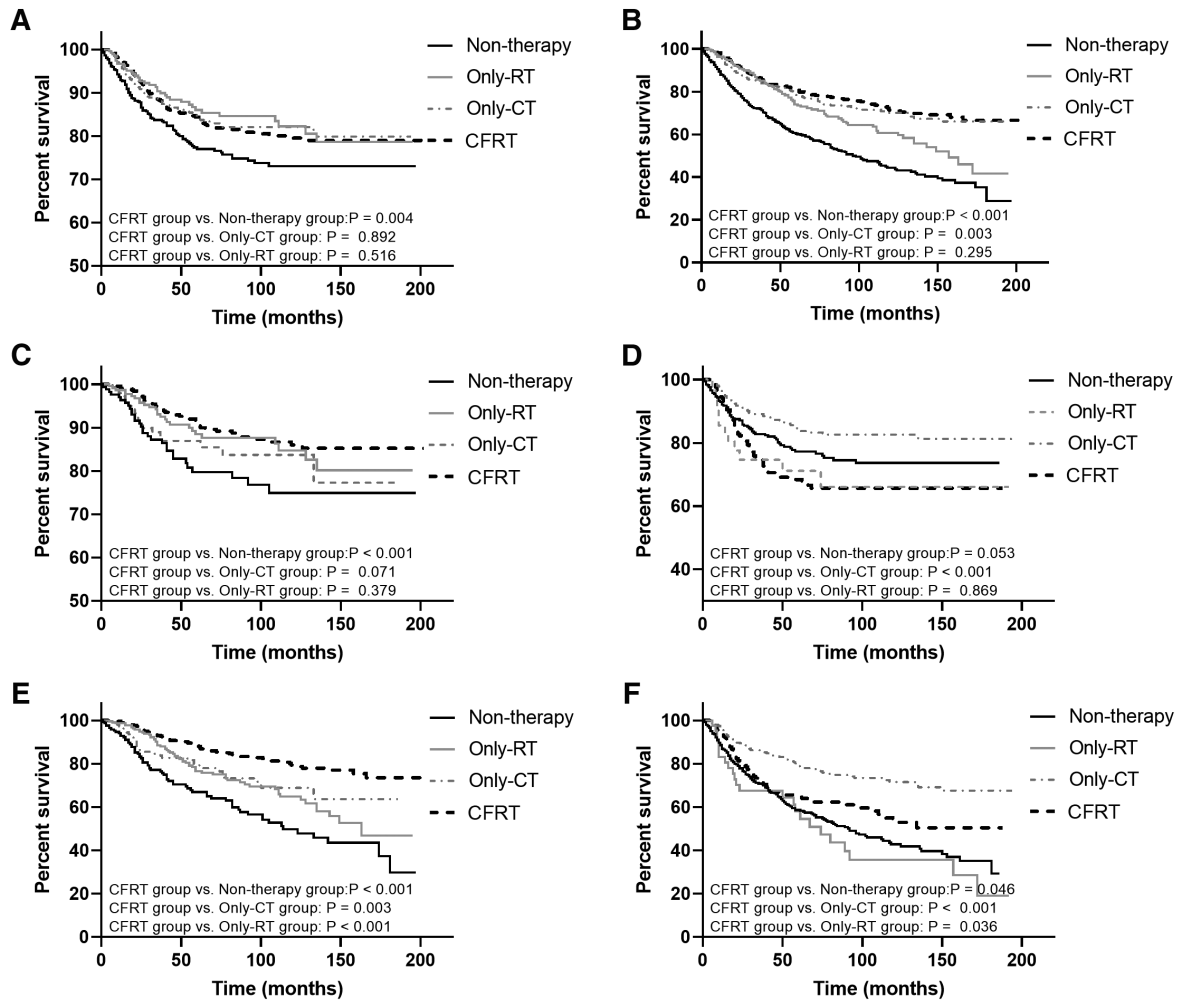
Prognosis of MBC patients with N0M0 received therapy. (A) BCSS of MBC patients; (B) OS of MBC patients; (C) BCSS of MBC patients received BCS; (D) OS of MBC patients received BCS; (E) BCSS of MBC patients received TM; (F) OS of MBC patients received TM. BCS = breast-conserving surgery, BCSS = breast cancer-specific survival, CT = chemotherapy, CFRT = chemotherapy followed by radiotherapy, MBC = metaplastic breast cancer, RT = radiotherapy, TM = total mastectomy.

To explore the role of CFRT in BCSS of patients with N0M0, we included surgery type to plot the survival curves using the Kaplan–Meier curve. Our study evaluated the role of CFRT in BCSS and OS of patients with N0M0 under different surgical types. In Kaplan–Meier analysis, among patients who have undergone TM, those receiving CFRT had superior survival compared to those not receiving therapy (CFRT group vs non-therapy group, *P* < .001), and compared to those receiving only-RT (CFRT group vs only-RT, *P* = .003), and compared to those receiving only-CT (CFRT group vs only-CT, *P* < .001) (Fig. 1E). However, neither in the entire cohort (CFRT group vs non-therapy group, *P* = .004; CFRT group vs only-RT, *P* = .892; CFRT group vs only-CT, *P* = .516, Fig. 1A) nor among patients who have undergone BCS (CFRT group vs non-therapy group, *P* < .001; CFRT group vs only-RT, *P* = .071; CFRT group vs only-CT, *P* = .379, Fig. 1C) could patients achieve better BCSS than those receiving other therapy types. Also, patients who underwent CFRT could not achieve better OS than those receiving other therapy types, either in the entire cohort (Fig. 1B) or in BCS (Fig. 1D) or in TM patients (Fig. 1F).

**Figure 2.**
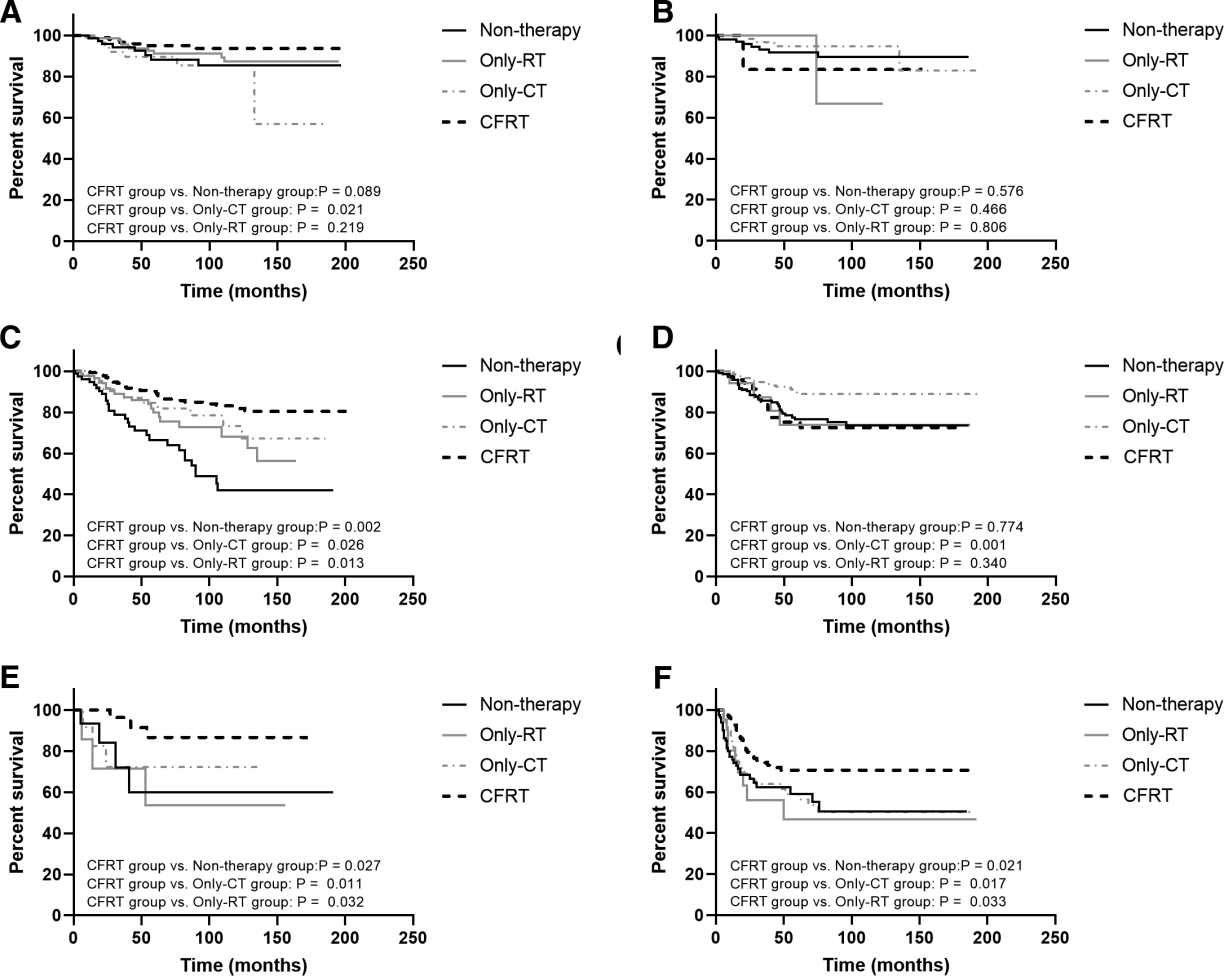
BCSS of MBC patients with N0M0. (A) Patients with stage T1 received BCS; (B) patients with stage T1 received TM; (C) patients with stage T2 received BCS; (D) patients with stage T2 received TM; (E) patients with stage T3 received BCS; (F) patients with stage T3 received TM. BCS = breast-conserving surgery, BCSS = breast cancer-specific survival, CT = chemotherapy, CFRT = chemotherapy followed by radiotherapy, MBC = metaplastic breast cancer, RT = radiotherapy, TM = total mastectomy.

Our study further evaluated the role of CFRT in patients with different tumor sizes. When patients underwent BCS, those who received CFRT had better BCSS and superior survival than other therapy types in stage T2 (CFRT group vs non-therapy group, *P* = .002; CFRT group vs only-RT, *P* = .026; CFRT group vs only-CT, *P* = .013, Fig. 2C) and T3 (CFRT group vs non-therapy group, *P* = .027; CFRT group vs only-RT, *P* = .011; CFRT group vs only-CT, *P* = .032, Fig. 2E), whereas patients with stage T1 could not achieve better BCSS than other therapy types (CFRT group vs non-therapy group, *P* = .089; CFRT group vs only-RT, *P* = .021; CFRT group vs only-CT, *P* = .219, Fig. 2A). However, when patients underwent TM, it was in stage T3 (CFRT group vs non-therapy group, *P* = .021; CFRT group vs only-RT, *P* = .017; CFRT group vs only-CT, *P* = .033, Fig. 2F) not in T1 (Fig. 2B) or in T2 (Fig. 2D) that those who received CFRT had better BCSS.

Additionally, our study also evaluated the role of CFRT in OS. When patients underwent BCS, those who received CFRT had better OS and superior survival compared to other therapy types in the entire cohort (all *P* < .05, Fig. S1A, S1C, and S1E, Supplemental Digital Content 2). However, when patients underwent TM, it was in stage T3 (all *P* < .05, Fig. S1F, Supplemental Digital Content 2) not in T1 (Fig. S1B, Supplemental Digital Content 2) or in T2 (Fig. S1D, Supplemental Digital Content 2) that those who received CFRT had better OS.

## 4. Discussion

In our study, we investigated how CT and RT impact the prognosis of N0M0 MBC and also looked into the potential benefits of CFRT. Our findings revealed that CFRT was beneficial for patients undergoing BCS only if they had stage T1 and T2 MBC. Similarly, for patients undergoing TM, CFRT was beneficial for those with stage T3 MBC.

The effect of breast cancer RT on MBC remains controversial. Jung et al^[13]^ performed a study with patients diagnosed with MBC from 2001 to 2008.

Their conclusion was that patients who underwent RT had no better disease-free survival and OS than those who did not undergo RT. Cecilia et al^[30]^ enrolled patients with stage I to III breast cancer identified from the National Cancer Database between 2010 and 2014. They concluded that MBC patients with stage I to III can benefit from RT. The reason for this effect could be the fact that, on the one hand, the sample size of the study varies greatly. On the other hand, different eras might yield different results. As pathologists’ understanding and surgeons’ recognition of MBC has been improved, the prognosis might have also been improved. Waqar et al found that In the lumpectomy cohort, the addition of RT was associated with improved OS in pT1–2N0 cases. This was more pronounced in pT3–4 or N+ cases. When analyzing these subcohorts in the mastectomy population, postmastectomy RT did not seem to influence OS in pT1–2N0 cases. However, postmastectomy RT for pT3–4 or N+ disease was associated with an OS benefit.

The proportion of patients who underwent CT ranged from 33 to 86%.^[12,17,31]^ On the one hand, patients undergoing extensive CT treatment may indicate that the efficacy of CT in patients with MBC is uncertain, although some small, single-institution, and single-arm studies have shown that CT can improve the prognosis of MBC.^[32–34]^ On the other hand, the high rate of patients receiving CT may be due to their tumors’ tendency to more common triple-negative phenotype and larger tumor size.^[35]^ Another reason is that, in the National Comprehensive Cancer Network guideline, the management strategy of MBC is paralleled to IDC.^[11]^ However, most studies report that CT might not affect the prognosis.^[2,13,15,16]^ In the present study, 60.4% of patients underwent CT, and it was associated with a better prognosis, which was consistent with the previous study.^[30,36]^

Due to the characteristics of the more triple-negative subtype, larger tumor size, and particular resistance to conventional systemic therapies, the treatment strategies provided by clinicians might be more radical. To improve the prognosis of patients, our study aimed to evaluate the effect of CFRT on MBC with N0M0. In the current literature, 28.4%, 31.3%, and 41.1% of patients received CFRT in stage T1, stage T2, and stage T3, respectively. However, only in stage T2 and T3 in the BCS group, and in stage T3 in the TM group, could CFRT improve the BCSS of MBC, according to our results. For OS, it is necessary for patients receiving BCS to perform CFRT regardless of tumor size. For patients receiving TM, CFRT had a beneficial outcome for MBC only in stage T3.

Our study has several limitations. First, due to its retrospective nature, it is characterized by observational bias and the possibility of selection bias. Second, the SEER database lacks the baseline characteristics of MBC patients, including work status, comorbidity, and socioeconomic environmental parameters. Third, this database cannot provide detailed CT and RT information, so it is impossible to conduct further case-control studies. However, our results will help researchers understand the role of CFRT in the prognosis of MBC.

## 5. Conclusion

Due to the characteristics of the more triple-negative subtype, larger tumor size, and particular resistance to conventional systemic therapies, the treatment strategies given by clinicians might be more radical. However, diligently and stringently evaluating chemotherapeutic and radiotherapeutic indications can avoid the waste of medical resources and reduce the suffering of patients.

## Acknowledgements

We sincerely thank the members of the Department for their strong support. Thanks to Dr Hu and Gong for their conception and design of this study and to Dr Zhang for her help with the methodology. During the period of writing and revising our manuscript, Dr Bao gave us many good suggestions, thanks sincerely.

## Author contributions

**Funding acquisition:** Jin Hu.

**Methodology:** Yun Bao.

**Project administration:** Lingxia Liao, Yiping Gong.

**Software:** Yanting Zhang.

**Supervision:** Lingxia Liao, Yiping Gong, Jin Hu.

**Writing – original draft:** Zankai Wu.

**Writing – review & editing:** Jin Hu.




