# Supplementary material for: A retrospective study of metaplastic breast cancer: Treatment and prognosis through chemotherapy followed by radiotherapy
Source: Medicine (Baltimore). 2026 Aug 7;105(32):e49401. doi: 10.1097/MD.0000000000049401 (PMC13456784; doi:10.1097/MD.0000000000049401)
Supplement: Supplementary file 1 [file medi-105-e49401-s001.docx]

| Supplementary Table 1. Prognostic factors for OS in MBC patients with N0M0. | | | | | | |
| --- | --- | --- | --- | --- | --- | --- |
| Variables | Univariate analysis | | P | Multivariate analysis | | P |
|  | HRs | 95% CI |  | HRs | 95% CI |  |
| Age | 1.044 | 1.038-1.0511 | < 0.001 | 1.036 | 1.028-1.034 | < 0.001 |
| Race/ethnicity (n, %) |  |  |  |  |  |  |
| Black | 1 | [Reference] |  | 1 | [Reference] |  |
| White | 0.911 | 0.730-1.137 | 0.409 | 0.825 | 0.658-1.033 | 0.093 |
| Other | 0.704 | 0.477-1.037 | 0.076 | 0.7227 | 0.491-1.077 | 0.112 |
| Insurance (n, %) |  |  |  |  |  |  |
| No | 1 | [Reference] |  | 1 | [Reference] |  |
| Yes | 1.137 | 0.954-1.354 | 0.152 | 1.043 | 0.870-1.251 | 0.649 |
| Grade (n, %) |  |  |  |  |  |  |
| Undifferentiated | 1 | [Reference] |  | 1 | [Reference] |  |
| Poorly differentiated | 0.606 | 0.443-0.829 | 0.002 | 0.609 | 0.444-0.836 | 0.002 |
| Moderately differentiated | 0.638 | 0.440-0.926 | 0.018 | 0.564 | 0.384-0.829 | 0.004 |
| Well differentiated | 0.376 | 0.217-0.650 | < 0.001 | 0.427 | 0.237-0.772 | 0.005 |
| Unknown | 0.870 | 0.614-1.233 | 0.433 | 0.619 | 0.433-0.885 | 0.008 |
| Histology (n, %) |  |  |  |  |  |  |
| Metaplastic carcinoma | 1 | [Reference] |  | 1 | [Reference] |  |
| Adenosquamous carcinoma | 0.618 | 0.399-0.956 | 0.031 | 0.757 | 0.471-1.217 | 0.250 |
| Carcinosarcoma | 1.065 | 0.701-1.618 | 0.769 | 1.029 | 0.675-1.571 | 0.893 |
| Others | 0.803 | 0.607-1.062 | 0.124 | 0.770 | 0.578-1.026 | 0.074 |
| Tumor size (n, %) |  |  |  |  |  |  |
| T1 | 1 | [Reference] |  | 1 | [Reference] |  |
| T2 | 1.572 | 1.273-1.942 | < 0.001 | 1.683 | 1.270-2.230 | < 0.001 |
| T3 | 3.917 | 3.077-4.987 | < 0.001 | 2.412 | 1.474-3.946 | < 0.001 |
| Surgery (n, %) |  |  |  |  |  |  |
| Non-surgery | 1 | [Reference] |  | 1 | [Reference] |  |
| BCS | 1.732 | 1.468-2.044 | < 0.001 | 1.052 | 0.866-1.276 | 0.611 |
| TM | 3.553 | 2.294-5.505 | < 0.001 | 3.454 | 2.167-5.506 | < 0.001 |
| Therapy types |  |  |  |  |  |  |
| Non-therapy | 1 | [Reference] |  | 1 | [Reference] |  |
| Only- RT | 0.590 | 0.462-0.754 | < 0.001 | 0.701 | 0.539-0.911 | 0.008 |
| Only-CT | 0.448 | 0.362-0.555 | < 0.001 | 0.604 | 0.479-0.763 | < 0.001 |
| CFRT | 0.394 | 0.321-0.483 | < 0.001 | 0.501 | 0.395-0.635 | < 0.001 |
| Abbreviations: OS = overall survival; MBC = metaplastic breast cancer; RT = radiotherapy; CT = chemotherapy; CFRT = chemotherapy followed by radiotherapy; BCS = breast conserving surgery; HRs = hazard ratios; CI = confidence interval; TM = Total mastectomy. | | | | | | |
